# Establishing the quality management baseline in the use of computed tomography machines in Kenya

**DOI:** 10.1120/jacmp.v13i1.3457

**Published:** 2012-01-05

**Authors:** Geoffrey K. Korir, Jeska S. Wambani, Ian K. Korir, Bernard O. Ochieng

**Affiliations:** ^1^ Department of Physics and Applied Physics University of Massachusetts Lowell Lowell MA 01854 USA; ^2^ Radiology Department Kenyatta National Hospital Nairobi Kenya; ^3^ National Nuclear Regulator Centurion 0157 South Africa; ^4^ Heart & Cancer Centre Aga Khan University Hospital Nairobi Kenya

**Keywords:** computed tomography, quality management, quality assurance, image quality

## Abstract

The objective of this study was to assess the level of compliance to quality assurance and image quality standards in computed tomography facilities in Kenyan hospitals. A quality assurance inspection and physical image quality assessment in eighteen representative computed tomography facilities were completed. A quantitative method was developed and used to score the results obtained from the physical image quality measurements using the American Association of Physicists in Medicine (AAPM) water phantom. Inspection was done in order to establish the level of compliance with internationally recognized standards such as those stipulated in the European Guidelines Quality Criteria for Computed Tomography and the International Basic Safety Standards for Protection against Ionizing Radiation. The overall findings placed the national quality management performance at 50±3%, while image quality and quality assurance performance were 61±3% and 37±3%, respectively. The quality assurance assessment benchmarked the country's level of quality management system compliance in diagnostic radiology. During accreditation appraisal, the scrutiny of scores from each stage in the medical imaging chain per facility will encourage continual implementation of the quality improvement process.

PACS number: 87.57.C, 87.57.cf, 87.57.cj, 87.57.cm, 87.57.cp, 87.57.Q, 87.55.N

## I. INTRODUCTION

Computed tomography (CT) is a medical X‐ray imaging modality that is now widely used. Numerous X‐ray beams are transmitted through the human body to obtain images for disease diagnosis, evaluation of clinical treatment, and assessment of patient wellness. The information obtained from transmission of the X‐ray beams through a three‐dimensional scanning of the body and the measurement of the amount by which the intensity is attenuated is digitally reconstructed to produce clinical images.^(^ ^1^ ^,^ ^2^ ^)^ This multistep processing of copious data, coupled with the performance of the device, technical expertise of operators, and other equipment factors, produce variability in patient dose in the application of CT scanners.

The European Commission (EC) has developed guidelines that include physical and anatomical image quality criteria in computed tomography.^(^ ^3^ ^)^ The use of the guidelines can support operational research in Radiology, as well as elevate the national health care management to global standards. The critical role of the Medical Practitioners and Dentist Board (MPDB) in regulating the health sector in Kenya is established. However, there is no reported use of any guidelines or other previous quality management (QM) studies conducted in diagnostic or therapeutic procedures in Kenya. Moreover, the protocols for establishing standards and guidelines in Radiology are yet to be adopted or implemented as recommended by the Kenya Association of Radiologists to the MPDB. The Radiation Protection Act, Cap 243 – Laws of Kenya, requires all irradiating devices in use in the country to comply with international safety standards.^(^ ^4^ ^)^ This law addresses regulatory requirements, but the acquisition and use of technologically advanced equipment is subject to the respective imaging professional societies, not to mention the dependence on cost, technical and human resource capabilities. To cope with these challenges, the Kenya Association of Radiologists is currently overseeing the collection of baseline data, and the development of quality systems and accreditation programs for diagnostic radiology departments. The International Atomic Energy Agency (IAEA), the Society of Radiographers in Kenya (SORK), and the Kenyatta National Hospital (KNH) are all contributing towards achieving this goal through a modular approach to quality assurance (QA) and image quality (IQ). Once established, it is envisaged that the services from the pioneering Medical Physics Department at KNH will benefit diagnostic departments in other hospitals in the country that are currently depending on quality control tests performed by the regulatory authority.

The complexity of CT scanner machines and the scanning techniques require a high level of training with respect to optimization, image quality, and shielding. This complexity makes the level of acceptance, equipment performance tests, and comprehensive equipment end user training essential to develop a national QM Standards. The standards will ensure conformity with the manufacturers' standards, and form the reference points for quality control tests and image quality criteria.^(^ ^3^ ^)^ Image quality assessment, therefore, is essential for: improving the efficacy of clinical interpretations, reducing the probability and magnitude of errors and repeated examinations, better utilization of economic resources, and better patient dose management. In a dynamic QA program, performance measures of optimization, device efficiency, and risks associated with high radiation exposures to patients can be assured. This study was aimed at establishing the initial baseline data and improvement of quality management performance in Kenya, with respect to image quality and quality assurance measures.

## II. MATERIALS AND METHODS

### A. General

This study reports the first prospective national project launched by the KNH, the Ministry of Health, and the IAEA. Structured questionnaire‐type forms were used during the necessary audits and inspections.

The CT scanners considered in the study were manufactured by well‐established multinational companies, namely Siemens (50%), General Electric (33%), Philips (11%), and Shimadzu (6%). The eighteen representative CT scanners out of the twenty‐one available in the country at the time of study were installed between 1987 and 2005. The physical image quality criteria were chosen in this investigation over anatomical quality factors due to the nonsubjectivity of the method. The fifteen quality assurance indicators used were: public safety, workers safety, quality control records, personnel monitoring, radiation signs, code of practice, patient records, patient preparation, patient shielding, in‐service training, QC technologist's skill level, service reports, quality assurance committee, professional certification, and device license. To enhance QC documentation and comparison with future test results, phantom images were adjusted with respect to the window level and window width, photographed, printed, and kept in the newly introduced QC paper files at each participating CT facility. The American Association of Physicists in Medicine (AAPM) performance phantom Model 76–410 QC testing equipment used in this study was donated by the IAEA through regional technical cooperation project RAF/9/033 Strengthening of Radiological Protection of Patients and Medical Exposure Control. In addition, a CT Dose Phantom Kit for Adult Head and Body was received following this study.

### B. Quality assurance assessment

The quality assurance assessment in this study involved quality factors chosen based on the organizational processes in the diagnostic department. The representative quality assurance criteria were selected from the EC,^(^ ^3^ ^)^ International Basic Safety Standards,^(^ ^5^ ^)^ and the Radiation Protection Act, Cap 243 Laws of Kenya.^(^ ^4^ ^)^ These factors were grouped into four categories, namely:

- The radiation safety consequences of operators' qualification, availability of relevant certifications, and use of protective gear.
- The performance and maintenance of the CT scanner, regarding relevant authorizations, servicing reports, presence of quality control programs, and record keeping.
- The availability, contents, and use of code of practice. This included quality assurance committees, training of personnel, appointment of quality control personnel, and all relevant quality aspects.
- The radiation protection measures, namely posting of radiation warning signs, monitoring of occupational exposure, and adequacy of shielding.

The presence or absence of implementation and standard documentation of the above quality factors led to the award of a score of one (pass) or zero (fail), respectively.

During the process of scanning the water phantom using head exposure protocols for the CT scanners evaluated, measurement of scatter radiation dose rates was done at strategic locations in the control room and uncontrolled area. Advanced Survey meter detecting gamma and X‐ray above 6 keV was chosen. The Victoreen with Pancake GM Probe Model 489–110D meter (Elimpex‐Medzintechnik GesmbH, Austria), with operating range of up to 800 μSv/hr, calibrated at an IAEA‐accredited regional Secondary Standards Dosimetry Laboratory (SSDL) in Arusha‐Tanzania. The shielding evaluation for each facility in the study was assessed for compliance with the indicated limitation of effective dose, as evaluated using Eq. (1):

(1) $$\left(\frac{s\times p\times t\times N\times T}{3600\;\text{sec}}\right)\leq 5\;\text{mSv}\;\text{per}\;\text{year}\;\text{or}\;1\;\text{mSv}\;\text{per}\;\text{year}$$

where *s* is the average scatter dose rate per slice at a specific area, *p* is the maximum number of examinations per year, *t* is the time in seconds per slice, *N* is the number of slices per examination, and *T* is the occupancy factor.^(^ ^6^ ^)^ The effective dose limits of 1 mSv or lower per year for the uncontrolled areas and 5 mSv or lower per year for controlled areas^(^ ^5^ ^)^ were applied in determining the award of a score of one (pass) or zero (fail).

### C. Image quality tests

#### C.1 Performance phantom description, preparation and assessment

IQ assessment was determined for each CT scanner using the AAPM water phantom described in Table 1. The results obtained from the standard methods in the water phantom user manual were assessed according to the test specifications shown in Table 2.

**Table 1:** Description of AAPM CT performance phantom.

*Name*	*Description*
Beam alignment insert	Aluminum pin, which is mounted axially on the inside of the phantom cover plate.
Slice thickness insert	Three 0.020"×1.00" aluminum strips angled at 45°, positioned on the center line and displayed vertically.
Linearity insert	Five 1“ diameter contrast pins of polyethylene, acrylic, polycarbonate, polystyrene, and nylon of densities indicated in Table 2.
High contrast resolution insert	8 sets of 5 hole cavities filled with air, spaced longitudinally on 4.3 mm centers and vertically on centers equal to twice the diameter.
Low contrast resolution extension	Long solid acrylic block having two each 2.25“ deep cavities of following diameters: 1”, 0.75“, 0.5”, 0.375“, 0.25”, and 0.125“. Spaced twice the appropriate diameter apart, one row of cavities on each side of the center line.

**Table 2:** European Guidelines test specifications for the CT physical image quality assessment.

*Performance parameter*	*Tolerance Specifications*
1 Beam alignment	Display of true image of the aluminium pin.
2 Noise	≤0.4% ^a^
3 CT No. accuracy	0±4 HU
4 CT No. uniformity	±8 HU
5 Polyethylene	−92 HU±5
6 Polystyrene	−24 HU±5
7 Water	0 HU±4
8 Nylon	+92 HU±5
9 Polycarbonate	+102 HU±5
10 Acrylic	+102 HU±6
11 Slice thickness	±25%
12 Size uniformity	30±1 cm
13 High spatial resolution	0.45−1.5 pairs per millimeter^(^ ^8^ ^)^
14 Low contrast resolution	<0.25″ diameter^(a)^

a This study derived tolerance limit. HU=Hounsfield units.

The image noise level was assessed using Eq. (2), which expresses the percentage of the effective linear attenuation coefficient of water corrected for the scanner contrast scale using acrylic and water:^(^ ^7^ ^)^

(2) $$S_{n}=\frac{100\times CS\times S}{\mu_{w}}$$

where *CS* is contrast scale, *S* is the estimated standard deviation of the CT number of picture elements in a specified area of the CT image, and $\mu_{w}$ is the linear attenuation coefficient of water. The image noise values obtained were scaled by ${\left(\frac{mAs}{200}\right)}^{1/2}$ in order to allow for comparison with the results obtained from all the CT scanners considered in the study. As indicated in Table 2, the physical image quality tests were evaluated for pass (1) or fail (0) according to the EC Guidelines,^(^ ^3^ ^)^ except for noise and contrast resolutions. The tolerance limits for noise, low contrast resolution, and high contrast resolution were not included in this guideline. To be objective, this study choose to use the maximum value from excellent performing CT scanners for noise and low contrast resolution tests limits, while the value reported by Papp^(^ ^8^ ^)^ was used as the tolerance limit for the high‐contrast resolution test. A total of fourteen points were available in the IQ compliance assessment.

The overall performance evaluated as QM (29 points) constitutes both QA (15 points) and IQ (14 points) assessment findings, and calculated as a percentage from the number of scores passed divided by the total of 29 quality factors considered. A score above or equal to 75% is rated as an achievement of excellent, 50%−74.9% is good, 30%−49.9% is fair, and less than 30% is poor. The average of all the CT facilities in the study was reported as the national QM performance level.

## III. RESULTS

### A. Quality assurance

**Figure 1:**
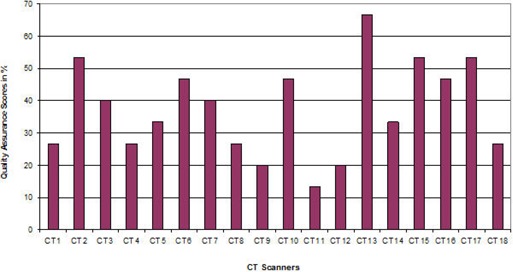
Quality assurance performance per CT scanner facility.

Figure 1 indicates the quality assurance performance results for each CT scanner facility with respect to all four categories stated above. According to the criteria used, none of the CT scanner facility scored above 75% or excellent, four were good, seven were fair, and seven were poor.

The contents in Table 3 indicate fair performance with respect to compliance with annual effective dose limits. In most facilities, the shielding integrity was compromised by lack of lead overlaps between the doors and door frames, as well as with the adjacent walls. In the control room, there was no lead equivalence between the observation window and the window frame.

**Table 3:** Estimated effective dose in mSv/yr with respect to the controlled and unrestricted areas for each CT scanner facility.

				*CT Scanners*					
	CT1	CT2	CT3	CT4	CT5	CT6	CT7	CT8	CT9
Public	5.5	2.0	0.7	0.8	0.2	0.3	1.0	0.5	0.6
Workers	2.6	6.3	1.2	0.1	6.8	0.6	0.3	1.0	5.5
				*CT Scanners*					
	CT10	CT11	CT12	CT13	CT14	CT15	CT16	CT17	CT18
Public	0.7	4.5	6	0.4	1.5	0.3	1.2	2.3	5.3
Workers	0.2	10.4	6.2	0.9	0.4	0.3	6.6	5.3	1.0

### B. Image quality

**Figure 2:**
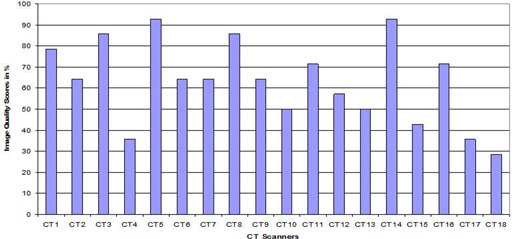
Image quality performance per CT scanner facility.

Figure 2 indicates the image quality performance. According to the criteria, five CT scanners achieved excellent image quality, seven were good, six were fair, and none was poor.

### C. Quality management

**Figure 3:**
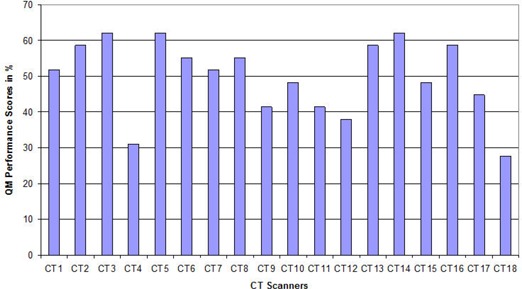
The quality management performance per CT scanner facility.

Figure 3 indicates the overall quality management performance. None of the CT scanner facilities scored above 75%, 10 were good, seven fair and one poor. Most CT scanners were accurate with respect to CT number uniformity (83%) and least accurate in high spatial resolution measurements (17%).

## IV. DISCUSSION

### A. Quality assurance

The CT scanner facility performance level on radiation safety (category I) indicated 41% of the facilities were compliant. The patient records were well kept, except the recording of patient radiation exposure which was displayed on the monitors of most CT scanners. All the imaging technologists operating the CT scanners were registered with SORK, the national imaging technologists' professional body. In most facilities, the use of protective gear over body areas outside the region of interest while scanning the patients was poor. There was a general assumption that the protective gear is only meant for the radiation workers and those who assist uncooperative or incapacitated patients. This finding indicated a low adherence to the use of basic protective gear, including the use of a breast garment of thinly layered bismuth impregnated with radio‐protective latex on the irradiated region.^(^ ^9^ ^,^ ^10^ ^)^

The equipment performance and maintenance level (category II) indicate 5% of the CT scanner facilities were compliant. The short and brief equipment service and maintenance reports were available in most facilities for billing purposes, but were inadequate to gain a passing score in this category. The engineering reports share a format similar to QC test reports; however, this study expected the former to be more comprehensive, as it is being carried out by technically competent personnel who are knowledgeable of equipment manufacturers' standards and detailed technical specifications requirements. The expected details, therefore, were not limited to equipment identification, details of service and maintenance, performance tests results, and relevant comments as to whether it was a pass or fail. During the study, only one CT facility provided a standard semiannual report, the information of which did not correspond with the results of this study conducted three months later. This result calls for regular performance of consistency and reproducibility checks to ensure that systematic errors are promptly detected and corrective actions applied.

The performance level in the use of code of practice (category III) indicated 9% of the CT scanner facilities were compliant. No facility fully sponsored in‐service training, officially appointed a QC technologist, established quality assurance committees, or kept adequate quality control records. A critical evaluation revealed divergent perspectives with respect to the contents and details of written code of practice, especially on the lack of standard methods of documenting patient preparation, nature of examination procedures, benefits and risks. However, the study had to infer safety measures being in place and practiced in accordance to the written documents. Therefore, stating duties and responsibilities clearly in relevant sections of the code of practice were treated as crucial.

The performance level in radiation protection measures (category IV) indicated 46% of the CT scanner facilities were compliant. In this study, the scrutiny of personnel radiation dose results was expected to yield information for identification of best practice, comparing with medical checkup, engineering controls, and calculation of collective dose. The resultant performance level was attributed to the lack of personnel trained in medical physics in the country. The absence of such qualified experts has also resulted in inadequate CT specification selection during procurement stage, absence of CT performance checks, dosimetry phantoms, and compliance audits.

When designing a room for CT scanner installation, the protection of personnel from radiation exposure is treated under radiation shielding. Additionally, the CT scanner gantries are equipped with shielding materials for primary and scatter radiation. Despite all these radiation protection measures, the results in Table 3 indicate only fair performance with respect to compliance with the effective dose limits for the staff and public. The need to promote training on appropriate shielding materials and methods is therefore necessary.

### B. Image quality

The performance with respect to beam alignment and slice thickness was 72% and 56%, respectively. Most CT scanners that failed the beam alignment test displayed an elliptical image of the aluminum pin and ring artifacts which are associated with detector element systems not producing proportional signals from the irradiation beam fan. The image noise measurements indicated 67% of the CT scanners had good calibration status and detector sensitivity. The wide range in image noise values obtained indicates the diversity in calibration status, as revealed by the linearity test. Good performance was noted in CT number uniformity (83%) which translates well to the expected CT numbers of water‐equivalent densities. The CT number uniformity test is comparable to the CT number accuracy obtained from one physical image test; however, the two cannot be used singly for routine QC tests because they require additional test to confirm linearity.

The linearity test performance indicated 28% of the CT scanners passed the test. Most CT scanners showed a shift of CT numbers in relation to the insert densities. These results are comparable to the IAEA reported values ^(^ ^11^ ^)^ and reflect the use of inbuilt calibration without routine QC checks. The linearity test is sensitive and appropriate for routine tests. When CT number deviations are observed, they can be normalized by use of tissue characterization and inhomogeneity correction.^(^ ^7^ ^)^ Unfortunately, as revealed in the quality assurance assessment, this may not be well accepted by most equipment operators who were found to have heavy workload and low technical expertise. A practical approach, therefore, is to use a more than two points system during calibrations, routine QC tests performance, and the use of better image processing computers.

The limiting high‐contrast spatial resolution results indicated the lowest performance (17%) of all the image quality criteria. These low performances require further investigation on the image reconstruction algorithms, detector performance, and configuration. When these variables are known, the EC guidelines propose contrast‐detail curve as an alternative method for evaluating this test.^(^ ^3^ ^)^ Comparatively, low contrast resolution results in the study revealed a good performance of 78% of the CT scanners as compared to the 17% for high contrast resolution. The tolerance level, therefore, could have been too high for the kind of CT scanners in the study for high contrast resolution, while the locally derived tolerance limit for low contrast resolution was reasonable.

### C. Quality management

Total quality management must also be promoted through departmental organization structure, standard operating procedures, optimal equipment performance, and application of internationally recognized Standards.^(^ ^12^ ^,^ ^13^ ^)^ The superior performance in image quality as compared to quality assurance in this study supports this philosophy, as well as the central aim of medical imaging of accomplishing diagnosis within a reasonable time, minimum equipment variables, and improved patient dose management. In contrast, the QA components constitute the QM elements that are dependent on cost, awareness and coordination of health administrators, imaging professionals, regulatory authorities and CT equipment vendors. Imaging professionals should therefore assert their roles and responsibilities in QM because they are inextricably linked to the quality of their product and service. Whereas the shared mission of quality patient care is noted, the results from this study also show the low interdependence of QA and IQ can be advanced if radiologists oversee the establishment of comprehensive QM systems, including effective QM committees, professional certification, quantitative measurements, equipment standards, imaging guidelines, technical capacity, and continuous professional development guided by application of recognized International Standards.

## V. CONCLUSIONS & RECOMMENDATIONS

This study has determined that quality management compliance in Kenyan medical imaging facilities is low. At the end of the study period, each participating CT facility received the evaluation report and the recommendations. Seminar presentations were prepared for imaging professionals and equipment suppliers, as well as hospital administrators. The implementation of the recommendations from this study may, however, be hampered by inadequate resources, staff expertise, time, and inadequate financial capability.

The QA factors that were inspected provide a basis for establishing a comprehensive QM program in the country. This assessment, however, was not exhaustive as there was poor performance in some of the quality factors considered. Hospitals were advised to collaborate with all the relevant professionals and stakeholders in establishing a comprehensive QA program.

This study indicated that the CT performance phantom is adequate in assessing CT scanners for radiation protection of patients. A QM committee should chose appropriate IQ criteria and establish the baseline data using clinical parameters for periodic QC testing.

The image noise results from this study reveal that by maintaining the image noise level of equal to or less than 0.4%, a good image quality is guaranteed. This image noise level is appropriate for consistency and compliance tests.

The method employed in assessing the radiation safety of the rooms installed with the CT scanners was sufficient, but the shielding partitions were inadequate. This outcome could be attributed to lack of knowledge of appropriate guidelines for radiation safety of medical CT facilities at the time of design and construction.

This study thus provides the baseline information that calls for urgent need for comprehensive QM through training, adequate regulations, professional certifications, clinical image quality guidelines, and accreditation of medical imaging facilities.

## ACKNOWLEDGMENTS

We sincerely thank the Ministry of Health, the Management and Radiology staff of all the health facilities for accepting to participate in the IAEA project (RAF/9/033‐Strengthening Radiological Protection of Patient and Medical Exposure Control), the University of Nairobi, the National Council for Science and Technology, and the International Atomic Energy Agency for their support.
